# Expression analysis of *PIN* family genes in Chinese hickory reveals their potential roles during grafting and salt stress

**DOI:** 10.3389/fpls.2022.999990

**Published:** 2022-09-29

**Authors:** Ying Yang, Jiaqi Mei, Juanjuan Chen, Ying Yang, Yujie Gu, Xiaoyu Tang, Huijie Lu, Kangbiao Yang, Anket Sharma, Xiaofei Wang, Daoliang Yan, Rongling Wu, Bingsong Zheng, Huwei Yuan

**Affiliations:** ^1^State Key Laboratory of Subtropical Silviculture, Zhejiang A&F University, Hangzhou, China; ^2^Zhejiang Provincial Key Laboratory of Forest Aromatic Plants-based Healthcare Functions, Zhejiang A&F University, Hangzhou, China; ^3^Research Institute of Subtropical Forestry, Chinese Academy of Forestry, Hangzhou, China

**Keywords:** *Carya cathayensis*, *PIN*, transport, auxin, salt, grafting

## Abstract

Grafting is an effective way to improve Chinese hickory while salt stress has caused great damage to the Chinese hickory industry. Grafting and salt stress have been regarded as the main abiotic stress types for Chinese hickory. However, how Chinese hickory responds to grafting and salt stress is less studied. Auxin has been proved to play an essential role in the stress response through its re-distribution regulation mediated by polar auxin transporters, including PIN-formed (PIN) proteins. In this study, the *PIN* gene family in Chinese hickory (*CcPINs*) was identified and structurally characterized for the first time. The expression profiles of the genes in response to grafting and salt stress were determined. A total of 11 *CcPINs* with the open reading frames (ORFs) of 1,026–1,983 bp were identified. Transient transformation in tobacco leaves demonstrated that CcPIN1a, CcPIN3, and CcPIN4 were localized in the plasma membrane. There were varying phylogenetic relationships between *CcPINs* and homologous genes in different species, but the closest relationships were with those in *Carya illinoinensis* and *Juglans regia*. Conserved N- and C-terminal transmembrane regions as well as sites controlling the functions of CcPINs were detected in CcPINs. Five types of *cis*-acting elements, including hormone- and stress-responsive elements, were detected on the promoters of *CcPINs*. *CcPINs* exhibited different expression profiles in different tissues, indicating their varied roles during growth and development. The 11 *CcPINs* responded differently to grafting and salt stress treatment. *CcPIN1a* might be involved in the regulation of the grafting process, while *CcPIN1a* and *CcPIN8a* were related to the regulation of salt stress in Chinese hickory. Our results will lay the foundation for understanding the potential regulatory functions of *CcPIN* genes during grafting and under salt stress treatment in Chinese hickory.

## Introduction

Auxin is the first discovered phytohormone (Went, 1935) and plays an important role during different growth stages of plants (Yu et al., 2022), including gametogenesis, seed germination, root elongation, vascular patterning, and blossoming (Zhao, 2010). In addition, an important function of auxin is its regulation of the responses of plant species to different biotic and abiotic stresses (Kapazoglou et al., 2020; Dastborhan et al., 2021; Mearaji et al., 2021; Zhang et al., 2022). The functioning of auxin is closely related to its homeostatic regulation, which involves biosynthesis, conjugate formation, transport, and degradation (Zhang and Peer, 2017). Following biosynthesis, transport is the key step for auxin functioning (Gomes and Scortecci, 2021) because the site of action is usually different from that of synthesis.

Plants have evolved two methods for transporting auxin: the long-distance network (transporting auxin through phloem) and the cell-to-cell transport network (also named polar auxin transport) (Muday and DeLong, 2001; Robert and Friml, 2009). In plants, polar auxin transport is mainly mediated by AUXIN/LIKE AUXIN proteins (AUX/LAX, responsible for auxin influx), PIN-formed proteins (PIN, responsible for auxin efflux), PIN-LIKES (PILS, responsible for auxin transport between the cytosol and endoplasmic reticulum), and ATP-binding cassette subfamily B proteins (ABCB, responsible for auxin influx/efflux) (Swarup and Peret, 2012; Mohanta et al., 2018; Sauer and Kleine-Vehn, 2019). PIN proteins play major roles in auxin efflux because their polar localizations are always consistent with the directionality of auxin (Zhou and Luo, 2018).

*PIN* is a gene family composed of multiple genes with similar or different structures and functions. *AtPIN1* was the first discovered PIN protein in *Arabidopsis*. The mutant of *PIN1*, *pin1*, has no leaves or flowers in cauline inflorescence (Kuhlemeier and Reinhardt, 2001) and resembles pins, which is the reason why PIN1 and the subsequent proteins in this family were named PIN-formed proteins (PINs). PIN family proteins contain conserved N- and C-terminal transmembrane domains and intracellular central hydrophilic loops. There are two types of PIN proteins categorized according to the differences in structures of the central hydrophilic loop: canonical PIN proteins with a long central hydrophilic loop and non-canonical PIN proteins with a short hydrophilic loop. In *Arabidopsis*, canonical PIN proteins (AtPIN1, 2, 3, 4, and 7) are localized to the plasma membrane (PM) and mediate auxin efflux to the extracellular space (Bennett et al., 2014). Non-canonical PINs (AtPIN5 and 8) are localized to the endoplasmic reticulum (ER) and mediate intracellular auxin transport between the cytoplasm and ER (Mravec et al., 2009; Sauer and Kleine-Vehn, 2019). AtPIN6 possesses a long hydrophilic loop and is located on the PM and ER, and thus is classified as a non-canonical PIN (Bennett et al., 2014).

The functions of PIN proteins have been well studied in *Arabidopsis*. AtPIN1 regulates the shoot apical meristem, root elongation, and the development of xylem (Blilou et al., 2005; Alabdallah et al., 2017). AtPIN2 influences root gravitropism and responds to salt stress (Wang et al., 2019; Gibson et al., 2020). AtPIN3 is mainly expressed in root and vascular tissues. It regulates auxin distribution in roots, participates in lateral root growth, and responds to light and gravity (Blilou et al., 2005; Zhai et al., 2021; Li et al., 2022). Similar to AtPIN3, AtPIN4 is related to light and gravity responses. In addition, AtPIN4 participates in auxin flow to the quiescent center (Bureau et al., 2010). AtPIN5 mediates not only the transport of auxin from the cytoplasm to the ER, but also regulates auxin homeostasis and metabolism (Mravec et al., 2009). AtPIN6 regulates lateral root formation (Sauer and Kleine-Vehn, 2019). The functions of AtPIN7 are similar to those of AtPIN3 and AtPIN4, and it participates in the gravimetric response of roots (Lewis et al., 2011). The function of AtPIN8 is the opposite of that of AtPIN5, transporting auxin from the ER to the cytoplasm (Ding et al., 2012). In addition, there is functional redundancy for some PIN proteins (Blilou et al., 2005; Wang et al., 2021). For example, the mutant genes *pin1* and *pin2* exhibit functional redundancy regarding the regulation of root meristem and root length (Vieten et al., 2005).

In addition to developmental functions, *PIN* family genes also participate in the regulation of abiotic stress (Yu et al., 2017). For example, in pepper (*Capsicum annuum*) and *Sorghum bicolor*, the expression profiles of *PIN* genes were changed under high salt treatment (Shen et al., 2010; Zhang et al., 2018). The process of grafting creates wounding stress, which also influences the expression of *PIN* genes (Melnyk, 2017; Sharma and Zheng, 2019). The overexpression of *AtPIN*s-*GUS* in *Arabidopsis* shows that *AtPINs* are expressed in the grafted union, indicating their involvement in grafting regulation (Wang et al., 2014). Transcriptome sequencing of *Arabidopsis* grafting also supports this conclusion (Melnyk et al., 2018).

Chinese hickory (*Carya cathayensis* Sarg.), belonging to the Juglandaceae family, is one of the most important trees economically in the Zhejiang and Anhui Provinces of China. Nuts from Chinese hickory trees are rich in nutritional compounds, including polyunsaturated fatty acids, phenolics, and flavonoids (Huang et al., 2022). However, a long juvenile phase and narrow distribution have restricted the development of the Chinese hickory industry. Grafting is an effective way to solve these problems. In addition, soil salinization has limited the development of the Chinese hickory industry.

To date, members of the *PIN* gene family have not been identified, and their potential roles during grafting and salt stress treatment in Chinese hickory are still unknown. In this study, *PIN* family genes in Chinese hickory were cloned and structurally analyzed, and their expression profiles in response to grafting and salt stress treatment were determined. Our results will lay the foundation for revealing the molecular mechanism of *PIN* family genes in regulating grafting and salt stress in Chinese hickory.

## Materials and methods

### Identification of *PIN* family genes in Chinese hickory

Candidate gene sequences of PIN in Chinese hickory (*CcPINs*) were identified from the published genome (Huang et al., 2019). The process used to identify genes was the same as that used in a previous study (Yang et al., 2021). Hidden Markov model (HMM) profiles of the PIN domain (PF03547) were used for analysis. TBtools (Toolbox for Biologists, v1.098745) was used for the preliminary screening of *PIN* genes (Chen et al., 2020). The identified *CcPINs* were named according to their phylogenetic relationships with the homologs in *Arabidopsis* (Supplementary Figure 1).

### Structural analysis of *CcPINs*

In addition to predicting the functions of *CcPINs* in Chinese hickory, the PIN proteins in Chinese hickory and 14 other species were chosen to undergo phylogenetic relationship analysis. The PIN proteins of 11 species were obtained from previous studies. PINs of the other three species (including *Juglans regia*, *Carya illinoinensis*, and *Nymphaea tetragona*) were analyzed based on the genome in Phytozome v13.^1^ The identified method was the same as that used for Chinese hickory, as stated above. The PIN protein information for 15 species is shown in Supplementary Table 1.

The phylogenetic tree between PIN proteins in the 15 species was constructed by MEGAX using the neighbor-joining method and a bootstrap of 1,000 replicates. ClustalW was used for multiple sequence alignment analysis of CcPINs. The motif distribution of CcPIN proteins was predicted by MEME Suite.^2^ The motif number was set to 8, and the maximum and minimum numbers of amino acids were set to 50 and 6, respectively. The online tool ExPASy was used to analyze biochemical information (length, molecular weight, isoelectric point, and size) of *CcPIN* genes. The transmembrane regions of CcPIN proteins were predicted by the TMHMM2 tool (TMHMM--2.0).^3^ ‘‘Search for CARE’’ on the PlantCare website^4^ was used to identify *cis*-acting regulatory elements on the promoters of *CcPIN* genes.

### Plant material, treatment, and sampling

One-year-old Chinese hickory seedlings were used for tissue-specific expression analysis, while 2-year-old Chinese hickory seedlings were used as rootstocks for grafting and materials for salt stress treatment. Seedlings were planted in seedling pots with a diameter of 17 cm and height of 22 cm and cultivated in the greenhouse of Zhejiang A&F University. One seedling was planted in each pot with 5 L of soil. The soil formula included 50% peat, 13% pastoral soil, 15% organic fertilizer, 5% exocarp, 10% agricultural bran, 5% perlite, and 2% release fertilizer. The seedlings were grown under the following conditions: temperature of 25 ± 3°C, humidity of 60–70%, and photoperiod of 12-h light/12-h dark. The seedlings were watered every 5 days. Tobacco (*Nicotiana benthamiana*) was cultivated in the culture room at the temperature of 22°C, humidity of 60–70%, and photoperiod of 16-h light/8-h dark. For subcellular localization analysis, 45-day-old tobacco leaves were used.

For the grafting experiment, 2-year-old seedlings with similar phenotypes were used for rootstocks, and the grafting operation was conducted on April 20th, 2018. One-year-old branches (7–8 cm in length) with a new bud collection on the fruit-bearing trees were chosen as scions. For salt stress treatment, the 2-year-old seedlings were treated with 150 mm NaCl (Shanghai Hushi Laboratorial Equipment Co., Ltd., China) solution three times every 3 days, following the same method as a previous study (Chen et al., 2015). The equivalent water treatment was used for the control check (CK).

Graft unions were collected at 0, 3, 7, and 14 days after grafting, representing the initiation stage, isolation layer formation stage, callus formation and isolating layer disappearance stage, and vascular bridge formation and linkage stage during Chinese hickory grafting, respectively, as detected by cytological observation (Liu et al., 2009). In each treatment, three graft unions were mixed together and regarded as a single sample. For salt stress treatment, samples from different tissues, including roots, stems, and leaves, were collected after 0, 1, 3, and 10 days of treatment, representing the initial-, short-, medium- and long-term treatment stages as reported in similar studies of other woody plants (Chen et al., 2015; Zhao et al., 2015; Hang et al., 2020; Huang et al., 2020). The day on which the seedlings were treated with NaCl solution for the first time was regarded as day 0. For each tissue, collections from five different seedlings were mixed together and regarded as a single sample. For tissue-specific expression analysis, different tissues, including roots, stems, leaves, and shoots, of 1-year-old Chinese hickory seedlings were collected in early spring (on April 16, 2022), representing the fast-growing stage of the seedlings. For each tissue, collections from five different seedlings were mixed together and regarded as a single sample. All the samples were wrapped in aluminum foil, immediately immersed in liquid nitrogen, and stored in an ultra-low temperature refrigerator (−80°C) for future use.

### RNA extraction, cDNA synthesis, and real-time quantitative PCR analysis

The total RNAs of Chinese hickory were extracted using a MiniBEST Universal RNA Extraction Kit [Code No. 9767, Takara Biomedical Technology (Beijing) Co., Ltd., China]. For the grafting experiment, the rootstocks and scions in a single sample were separated from each other, and the cells at the conjunction surfaces of the rootstocks and scions were scraped using blades, respectively. Total RNAs of the rootstocks and scions were extracted separately. For the salt stress experiment, the total RNAs of the roots, stems, and leaves collected at different time points were extracted separately. For the tissue-specific experiment, the total RNAs of the roots, stems, leaves, and shoots of 1-year-old seedlings were extracted separately. The cDNA used for cloning was synthesized using the PrimeScript™ IV 1st strand cDNA Synthesis Mix [Code No. 6215A, Takara Biomedical Technology (Beijing) Co., Ltd., China]. PrimeSTAR Max DNA Polymerase [Code No. R045Q, Takara Biomedical Technology (Beijing) Co., Ltd., China] was used for PIN gene cloning. The cDNA used for Real-Time Quantitative PCR analysis (qRT-PCR) was obtained using the PrimeScript™ RT Master Mix [Code No. RR036A, Takara Biomedical Technology (Beijing) Co., Ltd., China].

Real-Time Quantitative PCR analysis primers were designed on the National Center for Biotechnology Information (NCBI) primer prediction website.^5^ The *CcActin* gene was used as the internal standard for normalization. The primers for qRT-PCR are listed in Supplementary Table 2. The methods used for qRT-PCR were followed according to a previously published study (Yuan et al., 2018). The qRT-PCR analysis was replicated at least three times.

### Subcellular localization analysis

The coding sequence of *CcPINs* was cloned from the cDNA library of Chinese hickory and inserted into the modified pCAMBIA1300-GFP vector. Then, the obtained 35S:CcPINs-GFP was translated into *Escherichia coli* Trans1-T1 (TransGen Biotech Co., Ltd., China) and sequenced by Sangon Biotech (Shanghai) Co., Ltd., China. Plasmids from the identified strains were extracted and transformed into *Agrobacterium* strain GV3101 competent cells (AC1001, Shanghai Weidi Biotechnology Co., Ltd., China). Transient transformation of 45-day-old tobacco leaves was performed as previously described (Yamaji et al., 2009). The pm-rk (plasma membrane marker with red fluorescence, Nelson et al., 2007) was transiently co-transformed into tobacco leaves with *CcPINs*. After culturing for 48 h, the fluorescence signal in the transformed tobacco leaves was detected using a confocal microscope (LSM 880, Zeiss, Germany) with 488 and 594 nm argon lasers.

### Statistical analysis and figure preparation

To calculate the relative expression levels of *CcPIN* genes, the 2^–ΔΔ*CT*^ method, introduced by Schmittgen and Livak (2008), was used. A one-way analysis of variance (ANOVA) and multiple comparisons [the least significant difference (LSD) method] were conducted using SPSS Statistics (version 17.0) to analyze the expression differences of *CcPIN* genes among different tissues and treatments.

The histogram and line figures were drawn using Microsoft Excel 2019. The phylogenetic tree was drawn using Mega X and embellished using Fig. Tree (v1.4.4). The figures illustrating motifs and gene structures were drawn using TBtools (Toolbox for Biologists, v1.098745). Finally, the figures were merged by Adobe Photoshop (version 2020).

## Results

### Identification of *PIN* family genes in Chinese hickory

**FIGURE 1:**
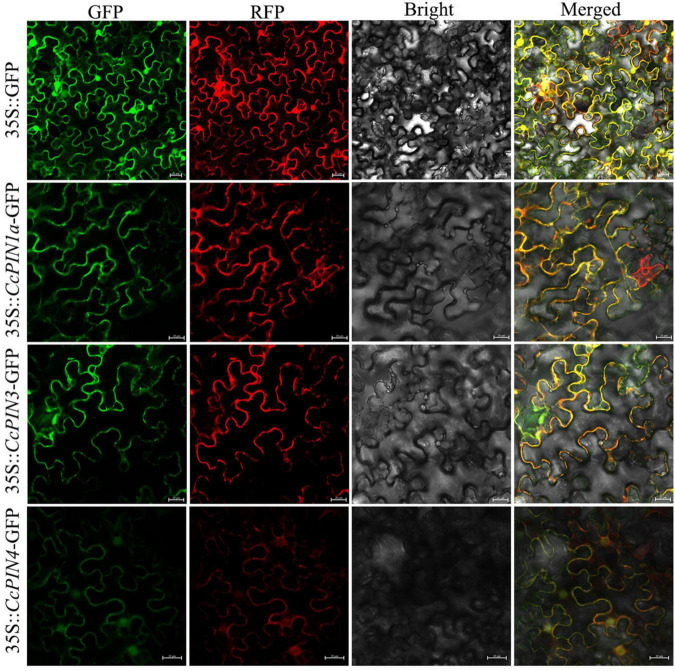
The subcellular localization of CcPIN1a, CcPIN3, and CcPIN4. The fluorescence images were captured in a dark field for green and red fluorescence, in a white field for the morphology of the cell, and in combination. GFP, green fluorescent protein fluorescence; RFP, red fluorescent protein fluorescence; Bright, bright field; Merged, GFP/RFP/bright field overlay. Bar = 20 μm.

Eleven *CcPIN* genes were identified, and the corresponding information is displayed in Table 1. The ORF length of the *CcPINs* ranged from 1,026 bp (CcPIN8a) to 1,983 bp (CcPIN3), encoding proteins with between 341 and 660 amino acids. The molecular weights of CcPINs ranged from 37,338.75 Da (CcPIN8a) to 72,046.05 Da (CcPIN3), and the predicted isoelectric points (pI) varied from 6.42 (CcPIN5) to 9.39 (CcPIN8a). The number of exons for *CcPIN* genes ranged from 5 to 7. To explore the potential locations where CcPINs perform their functions, several CcPINs were detected by subcellular localization. Transient transformation of *CcPINs* to tobacco leaves showed that CcPIN1a, CcPIN3, and CcPIN4 were expressed on the PM (Figure 1).

**TABLE 1:** Information on *CcPIN* genes and properties of their deduced proteins in Chinese hickory.

Gene name	Locus ID	Genomic location	Exon number	ORF (bp)	Length (aa)	pI	Mol wt (Da)	Ortholog with *Arabidopsis*
CcPIN1a	CCA1233S0022	scaffold50249:506539-510501	6	1,809	602	8.81	65,648.89	AtPIN1
CcPIN1b	CCA0709S0034	scaffold23721:408391-412462	6	1,779	592	8.95	63,882.92	AtPIN1
CcPIN2a	CCA0888S0033	scaffold31775:857604-860474	6	1,524	507	8.76	55,524.96	AtPIN2
CcPIN2b	CCA0674S0073	scaffold21864:981746-985490	6	1,926	641	9.22	69,513.49	AtPIN2
CcPIN3	CCA1410S0065	scaffold60275:585325-589367	6	1,983	660	7.69	72,046.05	AtPIN3
CcPIN4	CCA0857S0074	scaffold29914:809434-813085	6	1,932	643	8.45	70,068.83	AtPIN4
CcPIN5	CCA0888S0013	scaffold31775:259812-263129	5	1,086	361	6.42	39,945.26	AtPIN5
CcPIN6a	CCA0944S0041	scaffold35145:386749-392264	7	1,653	550	9.19	60,544.85	AtPIN6
CcPIN6b	CCA1391S0033	scaffold58974:378190-384204	7	1,629	542	9.26	59,537.80	AtPIN6
CcPIN8a	CCA0779S0095	scaffold26004:732921-735316	6	1,026	341	9.39	37,338.75	AtPIN8
CcPIN8b	CCA1094S0128	scaffold43199:1045218-1047610	6	1,104	367	6.46	40,253.74	AtPIN8

pI denotes isoelectric point, and Mol wt denotes molecular weight.

### Phylogenetic relationships of *PIN* family proteins in Chinese hickory and other species

**FIGURE 2:**
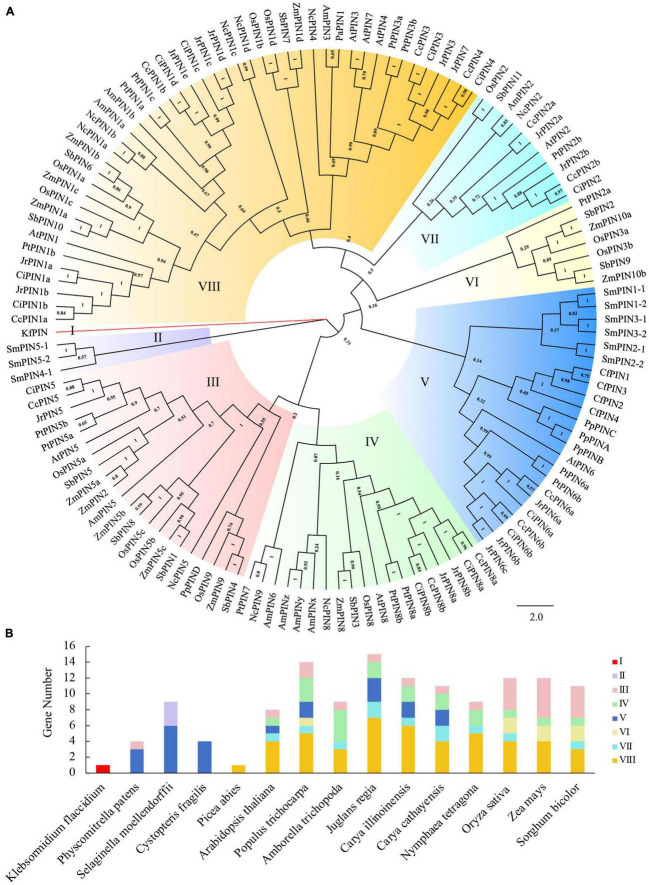
Phylogenetic analysis of PIN proteins from 15 species. **(A)** The phylogenetic tree. **(B)** The number and distribution of PINs in different species. Kf, *Klebsormidium flaccidium*; Pp, *Physcomitrella patens*; Sm, *Selaginella moellendorffii*; Cf, *Cystopteris fragilis*; Pa, *Picea abies*; At, *Arabidopsis thaliana*; Pt, *Populus trichocarpa*; Am, *Amborella trichopoda*; Jr, *Juglans regia*; Ci, *Carya illinoinensis*; Cc, *Carya cathayensis*; Nc, *Nymphaea tetragona*; Os, *Oryza sativa*; Zm, *Zea mays*; Sb, *Sorghum bicolor*.

To predict the potential functions of *PIN* family genes in Chinese hickory, a phylogenetic tree was constructed to explore the phylogenetic relationships between the 11 PIN proteins in Chinese hickory and 121 PIN proteins from 14 other plant species. The 15 plant species used to construct the phylogenetic tree belonged to 5 types of charophytes, bryophytes, lycophytes, monilophytes, gymnosperms, and angiosperms according to the consensus phylogeny reported by Doyle (2018). Detailed information on the 132 PIN proteins is shown in Supplementary Table 1. A total of eight groups (I, II, III, IV, V, VI, VII, and VIII) were identified (Figure 2A). There was significant variation in the number of PIN proteins in the different groups. There was only one protein in Group I, three proteins in Group II, seven proteins in Group VI, and 11 proteins in Group VII. The number of proteins in Groups III, IV, and V was 22, 19, and 23, respectively, and the highest number of proteins was in Group VIII, with 46 (Figure 2B).

The number of PIN proteins varied in different species. There was only one PIN protein in *Klebsormidium flaccidium* and *Picea abies*, while the number of PIN proteins in the other 13 species changed from 4 to 15 (Figure 2B). In addition, there were more PINs (8–15) in angiosperm species than those in the other types (Figure 2B). Similar to *C. illinoinensis* and *J. regia*, the PINs in *C. cathayensis* were distributed in Groups III, IV, V, VII, and VIII, with the largest number of PINs in Group VIII. Most PINs in *Oryza sativa*, *Zea mays*, and *S. bicolor* were distributed in Groups III, IV, VI, VII, and VIII (Figures 2A,B).

### Multiple sequence alignment, motif, and gene structure analysis of CcPINs

**FIGURE 3:**
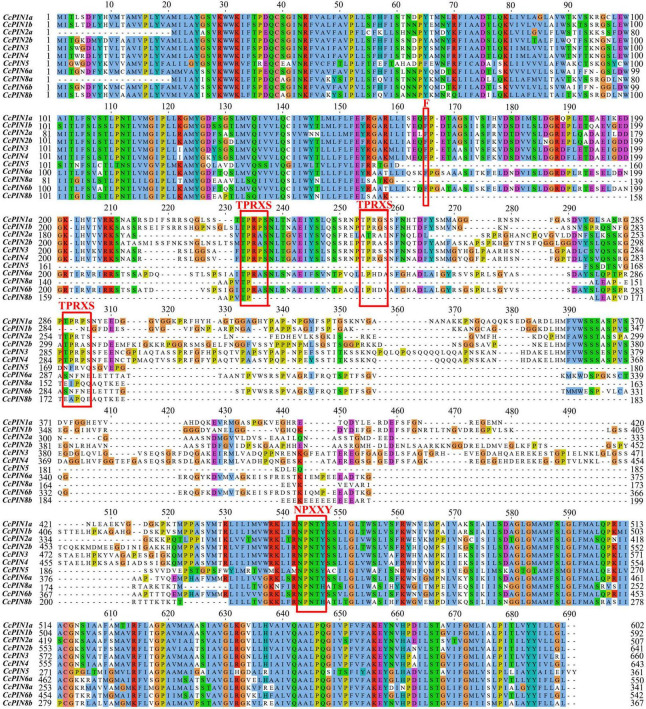
Multiple alignments of CcPINs by ClustalW. The possible functional sites are circled with red boxes.

**FIGURE 4:**
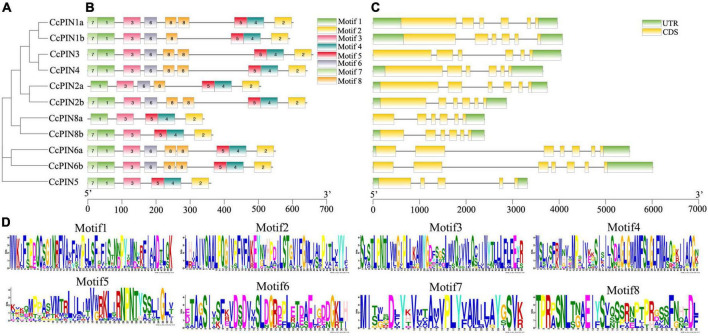
Motif and gene structure analysis of CcPINs. **(A)** Phylogenetic relationships. **(B)** Motif distribution. **(C)** Exon-intron structures. **(D)** Motif information.

Multiple sequence alignment of CcPIN proteins is shown in Figure 3, which displays the high level of conservation in the N-terminal and C-terminal regions. There were short sequences in the middle region for CcPIN5, CcPIN8a, and CcPIN8b, with long sequences for the other CcPINs. In addition, “F165,” the site controlling the distribution of PIN proteins on PM, the phosphorylation site “TPRXS,” and the conserved “NPXXY” site were detected (Figure 3). Motif analysis of the eleven CcPIN proteins was performed using the MEME Suite, with the eight conversed motifs set. Motif 6 and Motif 8 were not detected in short CcPINs (CcPIN5 and CcPIN8) (Figures 4A,B). The conserved element “NPXXY” was found in motif 5 (Figure 4D), and the phosphorylated site “TPRXS” of MPK 4/6 kinases was found in Motif 8 (Figure 4D). Transmembrane region prediction using TMHMM showed that there were N-terminal or C-terminal transmembrane regions for all CcPINs, with the total number ranging from 6 to 10 (Supplementary Figure 2). The coding sequences of *CcPINs* were compared with their corresponding DNA sequences on the genome. The numbers of exons in *CcPINs* ranged from five to eight (Figure 4C).

### Protein–protein interaction network prediction of CcPINs

**FIGURE 5:**
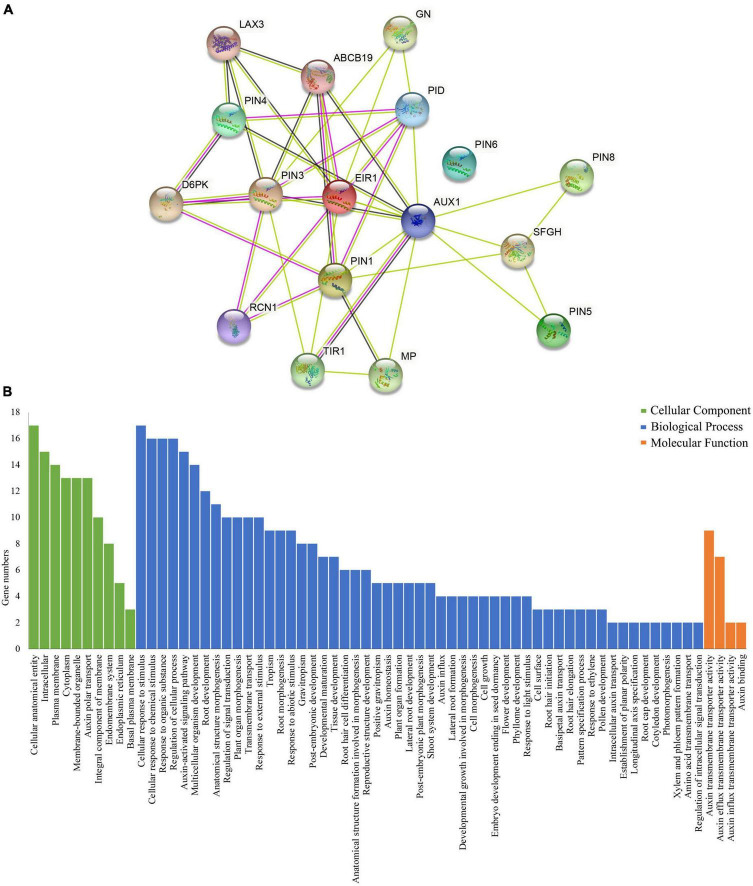
**(A)** QQProtein–protein interaction (PPI) network of PIN-formed (PINs) and **(B)** gene ontology (GO) annotation of proteins in the PPI.

The PPI network was predicted between the homologs of CcPINs in *Arabidopsis* and other proteins in *Arabidopsis* by the String database, with the minimum required interaction score set to a high confidence score of 0.700, and the maximum number of interactors was limited to no more than 10. The PPI network consisted of 17 nodes and 39 edges (Figure 5A). Seven nodes were representative of PIN proteins. The corresponding information is shown in Supplementary Table 3. Different line colors represent protein-protein associations.

The nodes were annotated using Gene Ontology (GO) (Figure 5B and Supplementary Table 4). The results showed that most of the predicted proteins contributed to biological processes, such as “Cellular response to stimulus” (GO:0051716), “Auxin-activated signaling pathway” (GO:0009734), “Tissue development” (GO:0009888), and “Xylem and phloem pattern formation” (GO:0010051). The proteins also played a role in multiple cellular components including the “Plasma membrane” (GO:0005886), “Auxin polar transport” (GO:0009926), and “Cytoplasm” (GO:0005737). In addition, the molecular function of the proteins included “Auxin transmembrane transporter activity” (GO:0080161), “Auxin efflux transmembrane transporter activity” (GO:0010329), “Auxin influx transmembrane transporter activity” (GO:0010328), and “Auxin binding” (GO:0010011).

### *Cis*-acting regulatory elements affecting the promoters of *CcPIN* family genes

**FIGURE 6:**
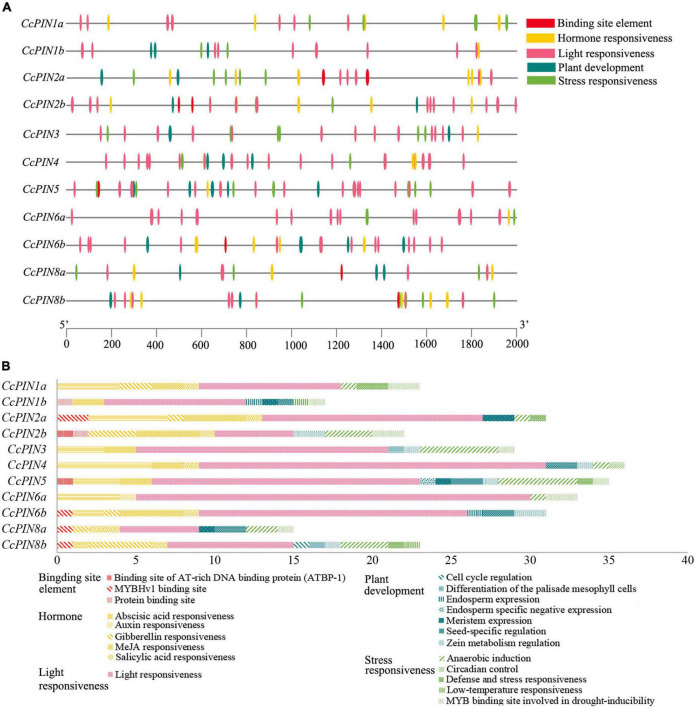
*Cis*-acting elements on the promoters of CcPIN family genes. **(A)** The distribution of *cis*-acting elements. **(B)** The number of *cis*-acting elements.

Regulatory elements that are *cis*-acting on the promoters of genes were the sites for expression regulation of genes. To explore how the expression of *CcPINs* was regulated by the different transcriptional factors, the section 2,000 bp upstream of the start codon (ATG) of *CcPINs* was used to query for *cis*-acting element detection using PlantCare. Five types of *cis*-acting elements, including a binding site element, hormone responsiveness, light responsiveness, plant development, and stress responsiveness, were detected, with numbers of 9, 71, 147, 28, and 40, respectively (Figure 6).

Within each type, specific *cis*-acting elements were detected. For example, five types of hormone-responsive elements, including gibberellin-responsive elements (GARE-motif, P-box, TATC-box, and TGA-element), abscisic acid-responsive elements (ABRE), MeJA-responsive elements (CGTCA-motif, TGACG-motif), and salicylic acid-responsive elements (TCA-element), were detected. The number of *cis*-acting elements for a certain *CcPIN* gene ranged from 15 (CcPIN8a) to 36 (CcPIN4), where light-responsive elements were the most abundant. In addition, five types of stress-responsive elements were detected on the promoters of *CcPINs*.

### Tissue-specific expression profiles of *CcPINs* in Chinese hickory

**FIGURE 7:**
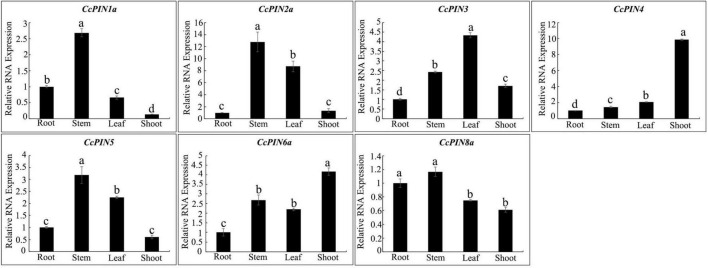
Tissue-specific expression profiles of *CcPIN* genes. Total RNA was extracted from roots, stems, leaves, and shoots of 1-year-old Chinese hickory. The relative expression levels of each *CcPIN* gene in roots were standardized as one. Different letters above the columns denote a significant difference in different tissues (*P* < 0.05).

To explore the relative expression of *PIN* genes in different tissues, tissue-specific expression profiles of *CcPIN* genes in roots, stems, leaves, and shoots of Chinese hickory were obtained by qRT-PCR. The results showed that *CcPIN1a*, *CcPIN2a*, *CcPIN5*, and *CcPIN8a* were highly expressed in stems, *CcPIN4* and *CcPIN6a* showed the highest expression levels in shoots, and the highest expression of *CcPIN3* was in leaves (Figure 7). The tissue-specific expression patterns of *CcPIN* genes showed that they might play different roles in different tissues in Chinese hickory.

### Expression profiles of *CcPIN* genes during grafting and salt stress in Chinese hickory

**FIGURE 8:**
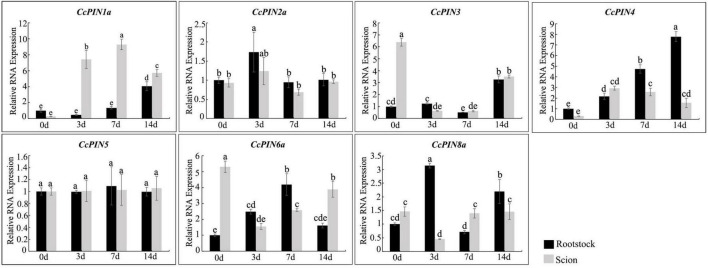
Relative RNA expression of *CcPIN* genes during the grafting of Chinese hickory. 0, 3, 7, and 14 d denote 0, 3, 7, and 14 days after grafting, respectively. The rootstocks collected at 0 day were regarded as the control sample. Different letters above the columns denote a significant difference in different treatments (*P* < 0.05).

To investigate whether *CcPINs* regulated grafting in Chinese hickory, the expression levels of *CcPINs* were measured at different stages of grafting (Figure 8). In scions, compared with 0 days after grafting, the expression level of *CcPIN1a* was significantly upregulated over sevenfold and eightfold at 3 and 7 days after grafting, respectively. However, at 14 days after grafting, the expression was decreased but still significantly higher than at 0 days after grafting. The expression of *CcPIN3* and *CcPIN6a* was downregulated in scions. The expression levels of *CcPIN4* and *CcPIN8a* significantly changed in rootstocks after grafting. The highest expression for *CcPIN4* was at 14 days after grafting in rootstocks, while *CcPIN2a* exhibited the highest expression at 3 days after grafting. *CcPIN5* showed stable expression during Chinese hickory grafting.

**FIGURE 9:**
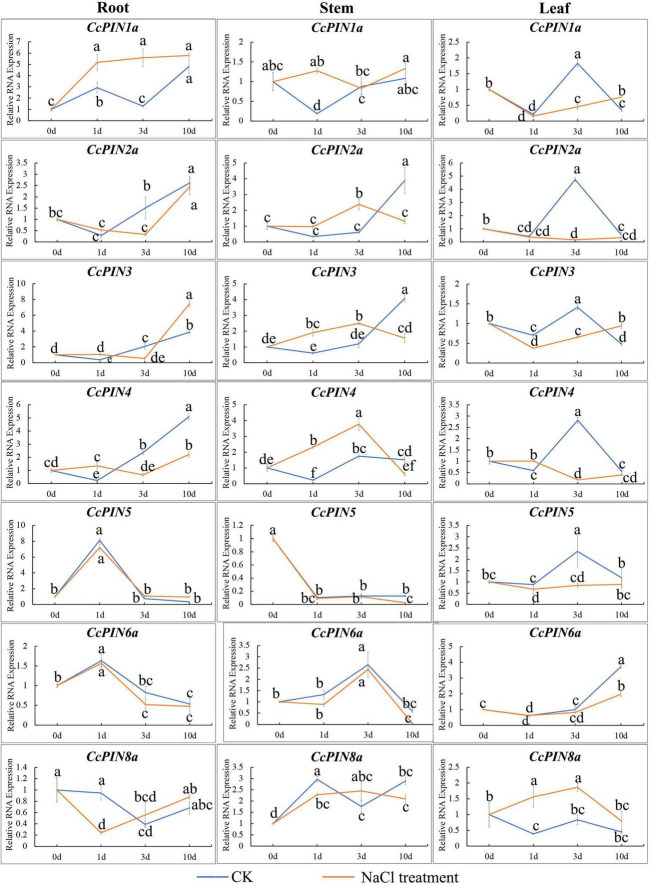
Relative RNA expression of *CcPIN* genes under CK (H_2_O) and salt stress treatments. 0, 1, 3, and 10 d represent 0, 1, 3, and 10 days after treatment, respectively. The sample from the CK group collected 0 days after treatment was regarded as the control sample. Different letters near the points indicate a significant difference in different treatments (*P* < 0.05).

To investigate how *CcPINs* respond to salt stress, the expression of *CcPINs* in the roots, stems, and leaves of Chinese hickory was determined at different time points in the salt stress treatment. *CcPINs* showed different expression profiles in different tissues (Figure 9). In roots, *CcPIN1a* was significantly upregulated after the salt stress treatment; *CcPIN2a*, *CcPIN3*, and *CcPIN4* were downregulated at 3 days after the salt stress treatment; and *CcPIN8a* was downregulated at 1 day after the salt stress treatment. In stems, *CcPIN1a*, *CcPIN3*, and *CcPIN4* were upregulated at 1 day after the salt stress treatment, while *CcPIN3* and *CcPIN4* were downregulated at 3 days after the salt stress treatment. The expression levels of *CcPIN5*, *CcPIN6a*, and *CcPIN8a* were similar to those after the CK treatment. In the leaves, most of the *CcPIN* genes were downregulated at 3 days after the salt stress treatment, while the expression of *CcPIN8a* was upregulated. The different expression profiles of *CcPINs* indicated that some of them might take part in the regulation of salt stress in Chinese hickory.

## Discussion

Chinese hickory is a native nut tree in China that is widely cultivated in Zhejiang and Anhui provinces. The nuts of Chinese hickory are not only rich in nutrition such as saturated and unsaturated fatty acids, proteins, essential amino acids for humans, and trace elements (Peng et al., 2013; Huang et al., 2022) but also have an important role in the medicinal field (Gao et al., 2020). High nutritional values have brought huge economic benefits for farmers. In 2021, the total output value of Chinese hickory in Lin’an distinct (Hangzhou, China) was 845 million RMB (∼123.3 million dollars) (Gu, 2022). However, the slow vegetative growth and narrow growing range have limited the development of Chinese hickory. Grafting is a technique that is commonly used in horticulture and has been applied to Chinese hickory to solve the difficulties in the Chinese hickory industry. In addition, it has been predicted that the salt in soil will be increased with increasing global temperatures (Dhankher and Foyer, 2018). Therefore, the study of grafting and salt stress in Chinese hickory is essential. PIN is an auxin efflux protein that participates in the regulation of plant grafting and salt stress (Wang et al., 2014; Chen et al., 2015). In the present study, we identified eleven PIN genes in Chinese hickory according to the published genome. The expression profiles in grafting and salt stress were determined to elucidate the function of *CcPINs*.

The number of identified *CcPIN* genes (11) was more than that in *Arabidopsis* (8). Phylogenetic analysis of 132 PIN proteins in 15 plant species was carried out to compare with *CcPINs*. On the phylogenetic tree, *CcPINs* were close to *CilPINs* in *C. illinoinensis*, which indicated that they might come from a common ancestor. To further study the similarity and diversity of *CcPIN* genes, an analysis of motif and intron/exon structure was executed. The exons of *CcPINs* were similar, and the various intron lengths were the main reason for different gene lengths, with similar results in other species (Wang et al., 2009; Hu et al., 2021).

PIN proteins play important roles in cellular auxin transport, and their function relies on corresponding sites in conserved regions (Zhou and Luo, 2018). In contrast, functional sites distributed in non-conserved regions might be unique in different species, and the phenomenon is related to sequence evolution (Zwiewka et al., 2019). In *Arabidopsis*, *PIN* genes consist of two conserved hydrophobic loops at the N- and C-terminus and a variable hydrophilic loop in the middle (Zhou and Luo, 2018). The analysis of multiple sequence alignments and motifs showed that there were similar motifs for *CcPINs*, and the motifs were distributed in a gene-conserved area. It was determined that F165 in AtPIN1 controls the polar distribution of the PIN protein (Sancho-Andrés et al., 2016). Functional F165 sites were also found in CcPIN proteins, indicating that they might perform the same function.

TPRXS, a phosphorylation site, appeared in motif 8 (Figure 3), and control of the polar distribution of PIN proteins was performed by phosphorylase kinase (Bassukas et al., 2022). The NPNXY site on the conserved domain is also related to PIN transport. Mutants of NPNXY in *AtPIN1* contributed to the accumulation of the PIN1 protein on the endoplasmic reticulum (Mravec et al., 2009). The multiple sequence alignment results show that all the CcPINs contained NPXXY in the conserved region, which suggests that the NPXXY motif performed the functional equivalent in Chinese hickory.

The plant hormone auxin regulates various developmental processes through its asymmetrical distribution on the PM (Vanneste and Friml, 2009). The different auxin distribution is executed by PINs (Wisniewska et al., 2006), and PIN-mediated auxin polar transport is crucial for auxin homeostasis. The PPI network predicted the multiple protein interactions with CcPINs through their homologous *AtPINs* genes. GO annotation showed that the predicted proteins were involved in many processes, and they participated in the regulation of auxin homeostasis together.

PINs are auxin efflux proteins that play important roles in plant life (Yu et al., 2022). To further explore the function of *CcPINs*, the expression levels from different tissues, including roots, stems, leaves, and shoots in Chinese hickory, were analyzed by qRT-PCR. The results showed that there was a high tissue-specific expression in the leaves, stems, and shoots of Chinese hickory, and the results were in accordance with those from previous studies. As previously reported, D6 PROTEIN KINASE and PINOID (PID)/WAG kinases activated auxin efflux transport PINs and influenced stem development (Wulf et al., 2019). In the woody plant Populus, *PtaPIN1* regulated stem growth, with high expression in the developing xylem (Carraro et al., 2012). *OsPIN5a* and *OSPIN5c* in rice were highly expressed in leaves. *PbPIN1*-3, *PbPIN2*-2, *PbPIN5*-1, and *PbPIN6* in pear trees were highly expressed in shoots (Qi et al., 2020). The tissue-specific transcriptional levels indicate that *CcPINs* participate in the regulation of hickory growth and development. Therefore, it is worthy of further research to clarify the detailed regulation process in Chinese hickory.

In the successful grafting of horticultural plants, the adhesion of different stems from rootstock and scion, namely the formation of the graft union, is necessary for grafting survival. During the process, callus formation and differentiation, and the reconnection of vascular tissues, are prominent (Melnyk et al., 2018; Sharma and Zheng, 2019). Auxin contributes to callus formation after a plant is wounded (Wulf et al., 2019). The imposed exogenous auxin also accelerates vascular reconnection (Wulf et al., 2019). In Chinese hickory grafting studies, morphological changes indicated that 3, 7, and 14 days after grafting were the crucial time points for isolation layer formation, callus formation, and vascular reconnection (Liu et al., 2009). Auxin-related genes, including *CcIAAs* and *CcGH3*, exhibited different expression levels at 0, 3, 7, and 14 days after grafting.

During *Arabidopsis* grafting, it has been proved that AtPIN1 participates in grafting with a high expression on the scion (Melnyk et al., 2018). To explore if auxin efflux proteins coding for *CcPIN* genes participated in the grafting process, the relative RNA expression of *CcPINs* was measured in rootstocks and scions at different time points by qRT-PCR. During the obtained results, the relative RNA expression level of *CcPIN1a* was upregulated 6–9 fold at the time points of 0, 3, 7, and 14 days after grafting. *CcPIN1a* is the ortholog of *AtPIN1*, indicating they might perform a similar function. Further study is necessary to elucidate the additional functions of *CcPIN1a* with respect to grafts. In addition to *CcPIN1a*, other *PIN* genes exhibited varied expression levels in grafting except for *CcPIN5*. The various expression profiles of *CcPIN* genes in different sections at the key stages of hickory grafting indicate their potential functions in grafting regulation.

When faced with salt stress, plants activate various mechanisms to resist the adverse environment. Auxin homeostasis and dynamic rearrangement are one of the regulatory pathways. A previous study showed that phospholipase D-derived phosphatidic acid combined with PID (PINOID/WAVY ROOT GROWTH) kinases enhanced the phosphorylation of PIN2, promoted auxin polar efflux ability, and contributed to the salt tolerance of *Arabidopsis* (Wang et al., 2019). *PIN1* and *PIN3* also participated in the process (Wang et al., 2019). During salt stress in watermelon, *ClPINs* performed functions of resisting salt with varying expressions (Yu et al., 2017). In the present study, samples of roots, stems, and leaves under salt stress were collected to explore the *CcPINs*’ expression profiles of anti-salt ability in Chinese hickory. As shown in Figure 9, the expression of CcPIN2a in roots and leaves was significantly downregulated, with variations similar to those in the previous report (Wang et al., 2019).

## Conclusion

To investigate the potential roles of auxin efflux transporters during stress treatments, eleven *CcPIN* genes with an ORF of 1,026–1,983 bp were identified in Chinese hickory for the first time, and their structural characteristics and expression responses to grafting and salt stress were detected by qRT-PCR. CcPINs were localized on the PM and had the closest relationships with homologous genes in *C. illinoinensis* and *J. regia*. Different hormone- and stress-responsive elements were detected on the promoter of *CcPINs*, indicating their expression regulation by stress treatments. *CcPINs* performed different functions during grafting and salt treatment. *CcPIN1a* has the potential to respond to grafting, while *CcPIN1a and CcPIN8a* might be involved in salt-stress regulation. In addition, *CcPINs* displayed different expression levels in different tissues, suggesting their varying roles during growth and development. Further investigations might be conducted on the mechanism of *CcPINs* for grafting and salt stress regulation.

## Data availability statement

The original contributions presented in this study are included in the article/Supplementary material, further inquiries can be directed to the corresponding authors.

## Author contributions

HY, BZ, XW, and DY conceived and designed the concept of the manuscript. YY (1st author), JM, JC, YY (4th author), and KY performed the experiments. YG, XT, HL, and HY analyzed the data. YY (1st author), JM, YG, and JC performed the formal analysis. YY (1st author), JM, and HY drafted the manuscript. AS, XW, DY, RW, BZ, and HY revised and finalized the manuscript. All authors endorsed the final version of the manuscript.

## Footnotes

1
https://phytozome-next.jgi.doe.gov/

2
https://meme-suite.org/tools/meme

3
https://services.healthtech.dtu.dk/service.php?TMHMM-2.0

4
http://bioinformatics.psb.ugent.be/webtools/plantcare/html/

5
https://www.ncbi.nlm.nih.gov/tools/primer-blast/

## References

[B1] Alabdallah O. Ahou A. Mancuso N. Pompili V. Macone A. Pashkoulov D. (2017). The *Arabidopsis* polyamine oxidase/dehydrogenase 5 interferes with cytokinin and auxin signaling pathways to control xylem differentiation. *J. Exp. Bot.* 68 997–1012. 10.1093/jxb/erw510 28199662

[B2] Bassukas A. E. L. Xiao Y. Schwechheimer C. (2022). Phosphorylation control of PIN auxin transporters. *Curr. Opin. Plant Biol.* 65:102146. 10.1016/j.pbi.2021.102146 34974229

[B3] Bennett T. Brockington S. Rothfels C. Graham S. Stevenson D. Kutchan T. (2014). Paralogous radiations of PIN proteins with multiple origins of noncanonical PIN structure. *Mol. Biol. Evol.* 31 2042–2060. 10.1093/molbev/msu147 24758777 PMC4104312

[B4] Blilou I. Xu J. Wildwater M. Willemsen V. Paponov I. Friml J. (2005). The PIN auxin efflux facilitator network controls growth and patterning in *Arabidopsis* roots. *Nature* 433 39–44. 10.1038/nature03184 15635403

[B5] Bureau M. Rast M. Illmer J. Simon R. (2010). *JAGGED LATERAL ORGAN (JLO)* controls auxin dependent patterning during development of the *Arabidopsis* embryo and root. *Plant Mol. Biol.* 74 479–491. 10.1007/s11103-010-9688-2 20852917

[B6] Carraro N. Tisdale-Orr T. E. Clouse R. M. Knoller A. S. Spicer R. (2012). Diversification and expression of the PIN, AUX/LAX, and ABCB families of putative auxin transporters in Populus. *Front. Plant Sci.* 3:17. 10.3389/fpls.2012.00017 22645571 PMC3355733

[B7] Chen C. Chen H. Zhang Y. Thomas H. R. Frank M. H. He Y. (2020). TBtools: An integrative toolkit developed for interactive analyses of big biological data. *Mol. Plant* 13 1194–1202. 10.1016/j.molp.2020.06.009 32585190

[B8] Chen Y. Cai J. Yang F. X. Zhou B. Zhou L. R. (2015). Ascorbate peroxidase from *Jatropha curcas* enhances salt tolerance in transgenic *Arabidopsis*. *Genet. Mol. Res.* 14 4879–4889. 10.4238/2015.May.11.20 25966262

[B9] Dastborhan S. Kalisz A. Kordi S. Lajayer B. A. Pessarakli M. (2021). “Physiological and biochemical responses of plants to drought and oxidative stresses,” in *Handbook of plant and crop physiology*, ed. Pessarakli M. (Boca Raton, FL: CRC Press), 517–541.

[B10] Dhankher O. P. Foyer C. H. (2018). Climate resilient crops for improving global food security and safety. *Plant Cell Environ.* 41 877–884. 29663504 10.1111/pce.13207

[B11] Ding Z. Wang B. Moreno I. Dupláková N. Simon S. Carraro N. (2012). ER-localized auxin transporter PIN8 regulates auxin homeostasis and male gametophyte development in *Arabidopsis*. *Nat. Commun.* 3:941. 10.1038/ncomms1941 22760640

[B12] Doyle J. A. (2018). Phylogenetic analyses and morphological innovations in land plants. *Annu. Plant Rev.* 45 1–50. 10.1002/9781119312994.apr0486

[B13] Gao F. Wu J. Zhou Y. Huang J. Lu J. Qian Y. (2020). An appropriate ratio of unsaturated fatty acids is the constituent of hickory nut extract for neurite outgrowth in human SH-SY5Y cells. *Food Sci. Nutr.* 8 6346–6356. 10.1002/fsn3.1623 33312521 PMC7723209

[B14] Gibson C. L. Isley J. W. Falbel T. G. Mattox C. T. Lewis D. R. Metcalf K. E. (2020). A conditional mutation in SCD1 reveals linkage between PIN protein trafficking, auxin transport, gravitropism, and lateral root initiation. *Front. Plant Sci.* 11:910. 10.3389/fpls.2020.00910 32733502 PMC7358545

[B15] Gomes G. L. B. Scortecci K. C. (2021). Auxin and its role in plant development: Structure, signalling, regulation and response mechanisms. *Plant Biol.* 23 894–904. 10.1111/plb.13303 34396657

[B16] Gu X. B. (2022). Small Chinese hickory nuts has brought economic benefits for industry development in Lin’an. *Zhejiang Forest.* 5 10–11.

[B17] Hang L. T. Mori K. Tanaka Y. Morikawa M. Toyama T. (2020). Enhanced lipid productivity of *Chlamydomonas reinhardtii* with combination of NaCl and CaCl2 stresses. *Bioprocess Biosyst. Eng.* 43 971–980. 10.1007/s00449-020-02293-w 32008095

[B18] Hu L. Wang P. Long X. Wu W. Zhang J. Pan Y. (2021). The PIN gene family in relic plant L. chinense: Genome-wide identification and gene expression profiling in different organizations and abiotic stress responses. *Plant Physiol. Biochem.* 162 634–646. 10.1016/j.plaphy.2021.03.030 33774468

[B19] Huang C. Li Y. Wang K. Xi J. Xu Y. Si X. (2022). Analysis of lipidomics profile of *Carya cathayensis* nuts and lipid dynamic changes during embryonic development. *Food Chem.* 370 130975. 10.1016/j.foodchem.2021.130975 34507207

[B20] Huang J. Wang S. Wang X. Fan Y. Han Y. (2020). Structure and expression analysis of seven salt-related ERF genes of Populus. *PeerJ.* 20:e10206. 10.7717/peerj.10206 33150090 PMC7583627

[B21] Huang Y. Xiao L. Zhang Z. Zhang R. Wang Z. Huang C. (2019). The genomes of pecan and Chinese hickory provide insights into *Carya* evolution and nut nutrition. *GigaScience* 8 giz036. 10.1093/gigascience/giz036 31049561 PMC6497033

[B22] Kapazoglou A. Tani E. Avramidou E. V. Abraham E. M. Gerakari M. Megariti S. (2020). Epigenetic changes and transcriptional reprogramming upon woody plant grafting for crop sustainability in a changing environment. *Front. Plant Sci.* 11:613004. 10.3389/fpls.2020.613004 33510757 PMC7835530

[B23] Kuhlemeier C. Reinhardt D. (2001). Auxin and phyllotaxis. *Trends Plant Sci.* 6 187–189. 10.1016/s1360-1385(01)01894-5 11335153

[B24] Lewis D. Negi S. Sukumar P. Muday G. (2011). Ethylene inhibits lateral root development, increases IAA transport and expression of PIN3 and PIN7 auxin efflux carriers. *Development* 138 3485–3495. 10.1242/dev.065102 21771812

[B25] Li R. Pan Y. Hu L. Yang D. Yuan M. Hao Z. (2022). PIN3 from liriodendron may function in inflorescence development and root elongation. *Forests* 13:568. 10.3390/f13040568

[B26] Liu C. Hong J. Xia G. Huang J. (2009). Cytological observation on healing responses in grafting of *Carya cathayensis*. *Sci. Silvae Sin.* 45 34–38.

[B27] Mearaji H. S. Ansari A. Igdelou N. K. M. Lajayer B. A. Pessarakli M. (2021). “Phytohormones and abiotic stresses: Roles of phytohormones in plants under abiotic stresses,” in *Handbook of plant and crop physiology*, ed. Pessarakli M. (Boca Raton, FL: CRC Press), 175–213.

[B28] Melnyk C. W. (2017). Plant grafting: Insights into tissue regeneration. *Regeneration* 4 3–14. 10.1002/reg2.71 28316790 PMC5350079

[B29] Melnyk C. W. Gabel A. Hardcastle T. J. Robinson S. Miyashima S. Grosse I. (2018). Transcriptome dynamics at *Arabidopsis* graft junctions reveal an intertissue recognition mechanism that activates vascular regeneration. *Proc. Natl. Acad. Sci. U.S.A.* 115 E2447–E2456. 10.1073/pnas.1718263115 29440499 PMC5878008

[B30] Mohanta T. K. Bashir T. Hashem A. Abd_Allah E. F. Khan A. L. Al-Harrasi A. S. (2018). Molecular players of auxin transport systems: Advances in genomic and molecular events. *J. Plant Interact.* 13 483–495. 10.1080/17429145.2018.1523476

[B31] Mravec J. Skupa P. Bailly A. Hoyerova K. Krecek P. Bielach A. (2009). Subcellular homeostasis of phytohormone auxin is mediated by the ER-localized PIN5 transporter. *Nature* 459 1136–1140. 10.1038/nature08066 19506555

[B32] Muday G. DeLong A. (2001). Polar auxin transport: Controlling where and how much. *Trends Plant Sci.* 6 535–542. 10.1016/s1360-1385(01)02101-x 11701382

[B33] Nelson B. K. Cai X. Nebenühr A. (2007). A multicolor set of in vivo organelle markers for co-localization studies in *Arabidopsis* and other plants. *Plant J.* 51 1126–1136. 10.1111/j.1365-313X.2007.03212.x 17666025

[B34] Peng Q. Bian W. Wang J. L. Wu J. Ge L. Y. Wang F. (2013). Effect of production techniques on nutrient content in Li’an pecans. *Sci. Technol. Food Ind.* 34 173–175. 10.13386/j.issn1002-0306.2013.20.056

[B35] Qi L. Chen L. Wang C. Zhang S. Yang Y. Liu J. (2020). Characterization of the auxin efflux transporter PIN proteins in pear. *Plants (Basel)* 9:349. 10.3390/plants9030349 32164258 PMC7154836

[B36] Robert H. Friml J. (2009). Auxin and other signals on the move in plants. *Nat. Chem. Biol.* 5 325–332. 10.1038/nchembio.170 19377459

[B37] Sancho-Andrés G. Soriano-Ortega E. Gao C. Bernabé-Orts J. M. Narasimhan M. Müller A. O. (2016). Sorting motifs involved in the trafficking and localization of the PIN1 auxin efflux carrier. *Plant Physiol.* 171 1965–1982. 10.1104/pp.16.00373 27208248 PMC4936568

[B38] Sauer M. Kleine-Vehn J. (2019). PIN-FORMED and PIN-LIKES auxin transport facilitators. *Development* 146:dev168088. 10.1242/dev.168088 31371525

[B39] Schmittgen T. D. Livak K. J. (2008). Analyzing real-time PCR data by the comparative CT method. *Nat. Protoc*. 3, 1101–1108. 18546601 10.1038/nprot.2008.73

[B40] Sharma A. Zheng B. (2019). Molecular responses during plant grafting and its regulation by auxins, cytokinins, and gibberellins. *Biomolecules* 9:397. 10.3390/biom9090397 31443419 PMC6770456

[B41] Shen C. Bai Y. Wang S. Zhang S. Wu Y. Chen M. (2010). Expression profile of PIN, AUX/LAX and PGP auxin transporter gene families in *Sorghum bicolor* under phytohormone and abiotic stress. *FEBS J.* 277 2954–2969. 10.1111/j.1742-4658.2010.07706.x 20528920

[B42] Swarup R. Peret B. (2012). AUX/LAX family of auxin influx carriers-an overview. *Front. Plant Sci.* 3:225. 10.3389/fpls.2012.00225 23087694 PMC3475149

[B43] Vanneste S. Friml J. (2009). Auxin: A trigger for change in plant development. *Cell* 136 1005–1016. 10.1016/j.cell.2009.03.001 19303845

[B44] Vieten A. Vanneste S. Wisìniewska J. Benkovaì E. Benjamins R. Beeckman T. (2005). Functional redundancy of PIN proteins is accompanied by auxin-dependent cross-regulation of PIN expression. *Development* 132 4521–4531. 10.1242/dev.02027 16192309

[B45] Wang J. Guo X. Xiao Q. Zhu J. Cheung A. Y. Yuan L. (2021). Auxin efflux controls orderly nucellar degeneration and expansion of the female gametophyte in *Arabidopsis*. *New Phytol.* 230 2261–2274. 10.1111/nph.17152 33338267 PMC8248126

[B46] Wang J. Jin Z. Yin H. Yan B. Ren Z. Z. Xu J. (2014). Auxin redistribution and shifts in PIN gene expression during Arabidopsis grafting. *Russ. J. Plant Physiol.* 61 688–696. 10.1134/s102144371405015x

[B47] Wang J.-R. Hu H. Wang G.-H. Li J. Chen J.-Y. Wu P. (2009). Expression of PIN genes in rice (*Oryza sativa* L.): Tissue specificity and regulation by hormones. *Mol. Plant.* 2 823–831. 10.1093/mp/ssp023 19825657

[B48] Wang P. Shen L. Guo J. Jing W. Qu Y. Li W. (2019). Phosphatidic acid directly regulates PINOID-dependent phosphorylation and activation of the PIN-FORMED2 auxin efflux transporter in response to salt stress. *Plant Cell* 31 250–271. 10.1105/tpc.18.00528 30464035 PMC6391703

[B49] Went F. W. (1935). Auxin, the plant growth-hormone. *Bot. Rev.* 1 162–182.

[B50] Wisniewska J. Xu J. Seifertová D. Brewer P. B. Ruzicka K. Blilou I. (2006). Polar PIN localization directs auxin flow in plants. *Science* 312 883–883. 10.1126/science.1121356 16601151

[B51] Wulf K. E. Reid J. B. Foo E. (2019). Auxin transport and stem vascular reconnection–has our thinking become canalized? *Ann. Bot.* 123 429–439. 10.1093/aob/mcy180 30380009 PMC6377096

[B52] Yamaji N. Huang C. F. Nagao S. Yano M. Sato Y. Nagamura Y. (2009). A zinc finger transcription factor ART1 regulates multiple genes implicated in aluminum tolerance in rice. *Plant Cell* 21 3339–3349. 10.1105/tpc.109.070771 19880795 PMC2782276

[B53] Yang Y. Huang Q. Wang X. Mei J. Sharma A. Tripathi D. K. (2021). Genome-wide identification and expression profiles of ABCB gene family in Chinese hickory (*Carya cathayensis* Sarg.) during grafting. *Plant Physiol. Biochem.* 168 477–487. 10.1016/j.plaphy.2021.10.029 34757298

[B54] Yu C. Dong W. Zhan Y. Huang Z. A. Li Z. Kim I. S. (2017). Genome-wide identification and expression analysis of ClLAX, ClPIN and ClABCB genes families in *Citrullus lanatus* under various abiotic stresses and grafting. *BMC Genet.* 18:33. 10.1186/s12863-017-0500-z 28388893 PMC5384148

[B55] Yu Z. Zhang F. Ding Z. (2022). Auxin signaling: Research advances over the past 30 years. *J. Integr. Plant Biol.* 2 371–392. 10.1111/jipb.13225 35018726

[B56] Yuan H. Zhao L. Chen J. Yang Y. Xu D. Tao S. (2018). Identification and expression profiling of the Aux/IAA gene family in Chinese hickory (*Carya cathayensis* Sarg.) during the grafting process. *Plant Physiol. Biochem.* 127 55–63. 10.1016/j.plaphy.2018.03.010 29549758

[B57] Zhai S. Cai W. Xiang Z. X. Chen C. Lu Y. T. Yuan T. T. (2021). PIN3-mediated auxin transport contributes to blue light-induced adventitious root formation in *Arabidopsis*. *Plant Sci.* 312:111044. 10.1016/j.plantsci.2021.111044 34620442

[B58] Zhang C. Dong W. Huang Z. A. Cho M. Yu Q. Wu C. (2018). Genome-wide identification and expression analysis of the CaLAX and CaPIN gene families in pepper (*Capsicum annuum* L.) under various abiotic stresses and hormone treatments. *Genome* 61 121–130. 10.1139/gen-2017-0163 29304291

[B59] Zhang H. Zhu J. Gong Z. Zhu J.-K. (2022). Abiotic stress responses in plants. *Nat. Rev. Genet.* 23 104–119. 10.1038/s41576-021-00413-0 34561623

[B60] Zhang J. Peer W. A. (2017). Auxin homeostasis: The DAO of catabolism. *J. Exp. Bot.* 68 3145–3154. 10.1093/jxb/erx221 28666349

[B61] Zhao R. Sun H. Zhao N. Jing X. Shen X. Chen S. (2015). The *Arabidopsis* Ca^2+^-dependent protein kinase CPK27 is required for plant response to salt-stress. *Gene* 563 203–214. 10.1016/j.gene.2015.03.024 25791495

[B62] Zhao Y. (2010). Auxin biosynthesis and its role in plant development. *Annu. Rev. Plant Biol.* 61 49–64. 10.1146/annurev-arplant-042809-112308 20192736 PMC3070418

[B63] Zhou J. J. Luo J. (2018). The PIN-FORMED auxin efflux carriers in plants. *Int. J. Mol. Sci.* 19 2759. 10.3390/ijms19092759 30223430 PMC6164769

[B64] Zwiewka M. Bilanovièová V. Seifu Y. W. Nodzyński T. (2019). The nuts and bolts of PIN auxin efflux carriers. *Front. Plant Sci.* 10:985. 10.3389/fpls.2019.00985 PMC6685051 31417597

