# Supplementary material for: Expression analysis of PIN family genes in Chinese hickory reveals their potential roles during grafting and salt stress
Source: Front Plant Sci. 2022 Sep 29;13:999990. doi: 10.3389/fpls.2022.999990 (PMC9557188; doi:10.3389/fpls.2022.999990)
Supplement: Supplementary file 5 [file Data_Sheet_1.docx]

Supplementary Material

# Supplementary Figures

## Supplementary Figure 1


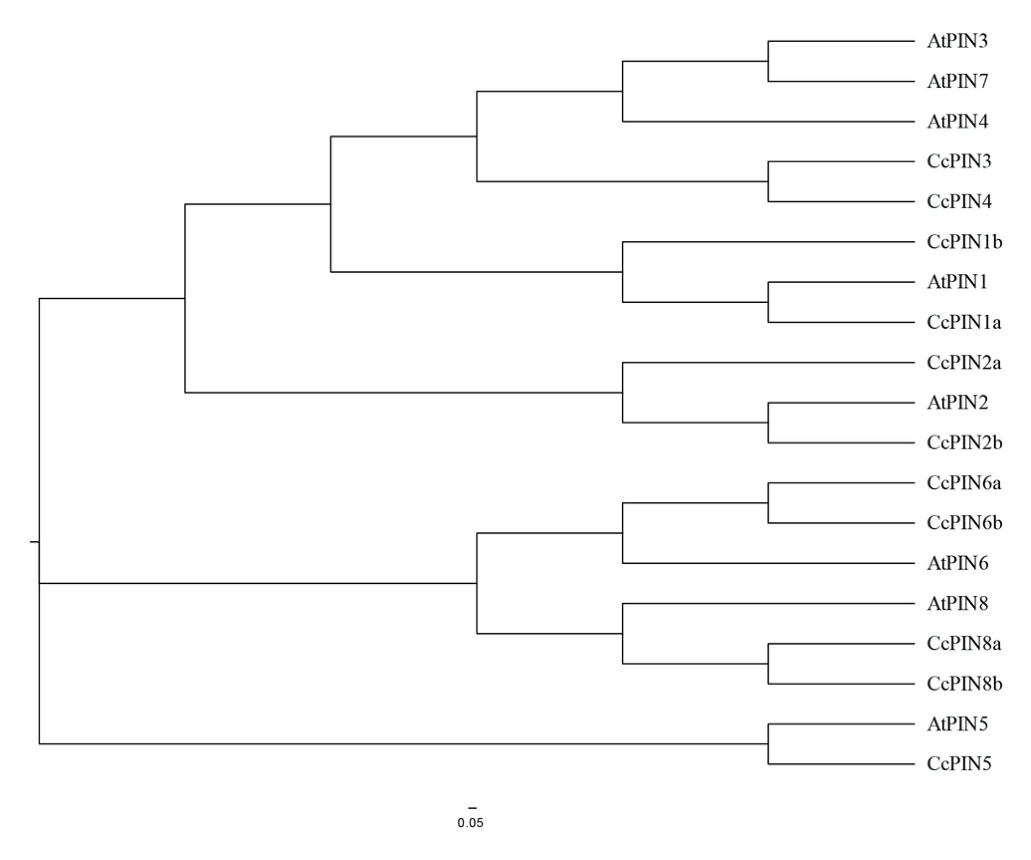


**Supplementary Figure** **1.** Phylogenetic analysis of AtPIN and CcPIN proteins. ClustalW was used for multiple sequence alignment analysis of AtPIN and CcPIN proteins. The phylogenetic tree was constructed by MEGAX with the neighbor-joining method and bootstrap of 1000 replicates.

## Supplementary Figure 2


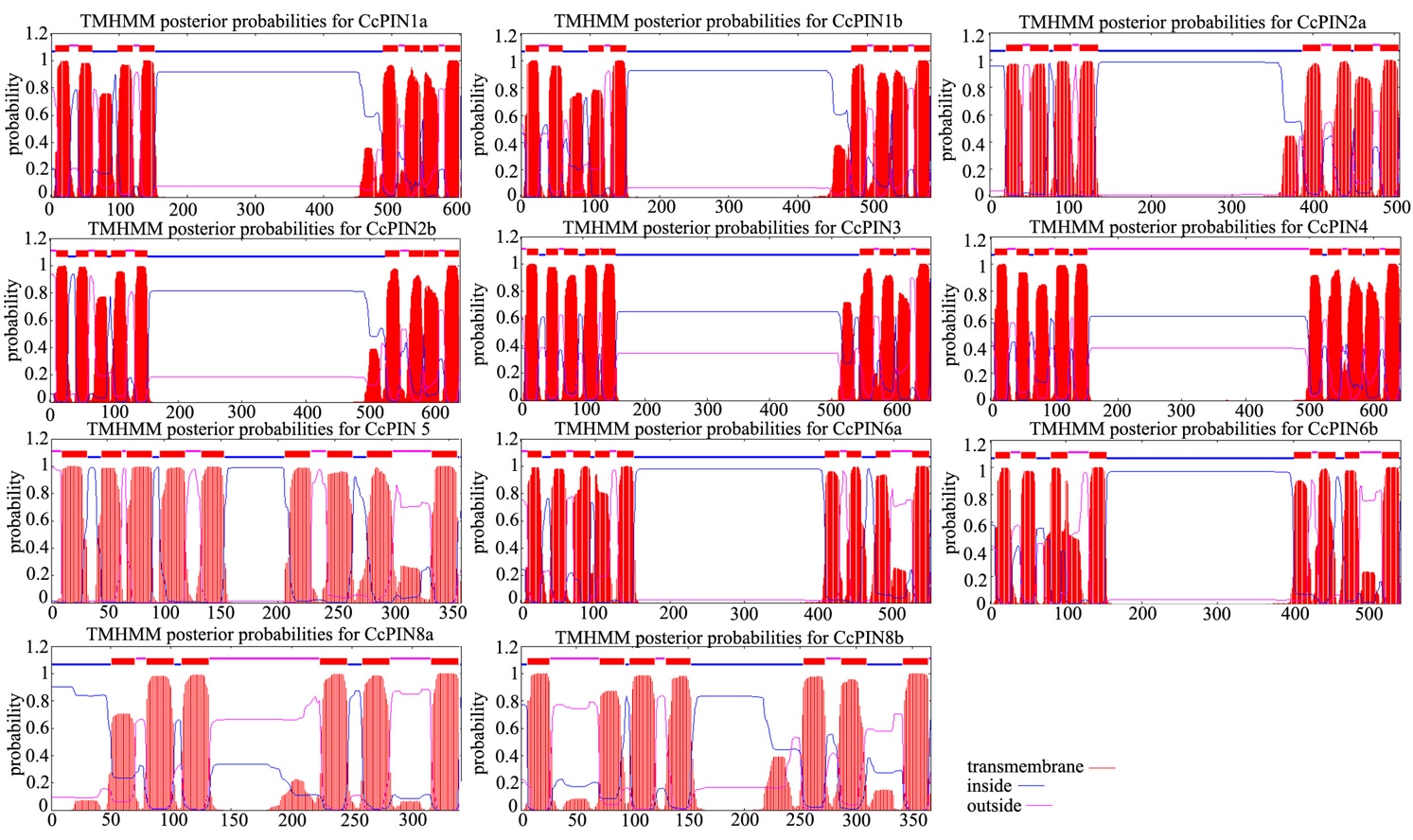


**Supplementary Figure 2.** Predicted transmembrane regions of CcPIN proteins through TMHMM2 tool. The predicted transmembrane helices were exhibited as red peaks on the top.
